# Molecular characterization and *in vitro* differentiation of feline progenitor-like amniotic epithelial cells

**DOI:** 10.1186/scrt344

**Published:** 2013-10-30

**Authors:** Lucia Rutigliano, Bruna Corradetti, Luisa Valentini, Davide Bizzaro, Aurora Meucci, Fausto Cremonesi, Anna Lange-Consiglio

**Affiliations:** 1 Department of Emergency and Organ Transplantations, University of Bari “Aldo Moro”, Bari, Italy; 2 Department of Life and Environmental Sciences, Università Politecnica delle Marche, Ancona, Italy; 3 Large Animal Hospital, Reproduction Unit, Università degli Studi di Milano, Lodi, Italy; 4 Department of Veterinary Science for Animal Health, Production and Food Safety, Università degli Studi di Milano, Milano, Italy

## Abstract

**Introduction:**

While amniotic mesenchymal cells have been isolated and characterized in different species, amniotic epithelial cells (AECs) have been found only in humans and horses and are recently considered valid candidates in regenerative medicine. The aim of this work is to obtain and characterize, for the first time in the feline species, presumptive stem cells from the epithelial portion of the amnion (AECs) to be used for clinical applications.

**Methods:**

In our study, we molecularly characterized and induced *in vitro* differentiation of feline AECs, obtained after enzymatic digestion of amnion.

**Results:**

AECs displayed a polygonal morphology and the mean doubling time value was 1.94 ± 0.04 days demonstrating the high proliferating capacity of these cells. By RT-PCR, AECs expressed pluripotent (*Oct4*, *Nanog*) and some mesenchymal markers (*CD166*, *CD44*) suggesting that an epithelial-mesenchymal transition may occur in these cells that lack the hematopoietic marker *CD34*. Cells also showed the expression of embryonic marker SSEA-4, but not SSEA-3, as demonstrated by immunocytochemistry and flow cytometry. Moreover, the possibility to use feline AECs in cell therapies resides in their low immunogenicity, due to the absence of *MHC-II* antigen expression. After induction, AECs differentiated into the mesodermic and ectodermic lineages, demonstrating high plasticity.

**Conclusions:**

In conclusion, feline AECs appear to be a readily obtainable, highly proliferative, multipotent and non-immunogenic cell line from a source that may represent a good model system for stem cell biology and be useful in allogenic cell-based therapies in order to treat tissue lesions, especially with loss of substance.

## Introduction

The main applications of mesenchymal stem cells (MSCs) in human medicine are in the therapy of hematological disorders, cardiovascular degenerative diseases, genetic and neurological disorders, and in tissue engineering [1], but to date there are few clinical advances in other pathologies. Two essential factors are necessary to promote the study in regenerative medicine: a good animal model and an efficient source of stem cells.

Since many pathologies are very difficult to study in human medicine, the domestic cat could offer an attractive animal model in order to explore different diseases with similarities to the human ones, as well as hereditary conditions (for example, autosomal dominant polycystic kidney disease) [2], hereditary retinal blindness [3], inherited muscular dystrophy [4], Niemann-Pick disease type C [5], diabetic neuropathy [6], immunodeficiency or viral diseases [7,8]. Moreover, since the cat genome project is nearly complete, the establishment of pluri/multipotent feline stem cells would facilitate targeting specific genetic loci, and generating additional useful disease models in the cat itself [9].

Regarding the stem cell reservoirs, the most characterized sources of MSCs are bone marrow (BM) [10-17] and the adipose tissue [12,17]. Also, in 2002, MSCs from BM in the cat were isolated for the first time and these cells appeared to be very similar to those obtained from rodent and human sources [18], but the procedures employed to isolate these tissues are invasive and cells are usually obtained with low efficiency [18-20].

Extra-fetal tissues could offer the possibility of getting over the limitations of adult stem cell sources [1,21-23]. Indeed, umbilical cord blood, umbilical cord matrix, amnion and amniotic fluid could provide a large amount of cells without risks for the donor and in an inexpensive and non-invasive way, since they are discarded at delivery, or can also be collected after cesarean section or in case of ovario-hysterectomy of pregnant uteri. This is a great concern for regenerative medicine, especially if there is the chance to cryogenically bank them [24,25].

Among extra-fetal tissues, recently, amniotic membrane appeared an important stem cell source in different species, including human [26], horse [23,27], sheep [28], dog [29] and cat [30]. The amniotic epithelium layer, while originating from the trophectoderm as other parts of fetal membranes, has the peculiarity of being continuous with the epiblast [31]. For this reason it may probably preserve some of the characteristics of the epiblast, like pluripotency [32], as confirmed by the expression of different pluripotent stem cell-specific transcription factors, such as *Sox2*, *Nanog*, *Oct4* and *Rex1*[27,32-36]. Amniotic epithelial cells (AECs) have been isolated and characterized in different species, such as in the human [36], horse [27] and sheep [37] and their pluripotency and plasticity are demonstrated by *in vitro* differentiation into the cell lines of the three germ layers [21,26,27,32,33,38-40]. The potential application of AECs in cell-based therapies relies not only on their pluripotent features, but also on their immunogenic characteristics. In fact, they do not express Major Histocompatibility Complex (MHC) Class II antigens [21,27,41,42]. In addition, AECs actively secrete a number of immunosuppressive factors with a consequent failure of allogeneic lymphocyte responsiveness, which may support survival following transplantation and engraftment [21,39,41-44].

The chance to characterize feline stem cells could be helpful in cell-based therapies in human medicine for the pathologies described above, but also in feline species to treat tissue lesions especially characterized by loss of substances. Moreover, these cells could also improve the efficiency of interspecies somatic cell nuclear transfer for preserving endangered felids [45] and could be used in drug testing in therapeutic intervention, and auto/allo/xenogenic transplantation.

Considering the reported context, in this study we isolated and characterized, for the first time, in terms of morphology, specific stemness and pluripotent markers, proliferative and differentiative potential, the AECs from the domestic cat.

## Materials and methods

### Amnion collection

Uteri were recovered from three pregnant queens at 40 to 45 days of gestational age, brought to the veterinary hospital by their owners to be spayed. In this study, after approval by the Ethical Committee of the University of Milan and the owner’s written consent, all procedures were conducted following standard veterinary practice and in accordance with 2010/63 EU directive on animal protection and Italian Law (D.L. No. 116/1992). According to the ethical guidelines, during ovariohysterectomy, care was given to maintain fetuses in the depressed state from maternal anesthesia until vital signs (heart beats) disappeared, as assessed by intra-surgical ultrasonography, before removal of the gravid uterus.

Samples of allanto-amnion were kept in a 4°C in phosphate-buffered saline (PBS) for not more than 24 h. The amniotic membrane of each sample was mechanically separated from the allantois and was cut into small pieces before enzymatic digestion to isolate presumptive AECs.

### Isolation of amniotic epithelial cells and cell culture

Amnion fragments were washed twice in Hank’s Balanced Salt Solution (Euroclone, Milano, Italy. ECB-4007 L) supplemented with penicillin 100 UI/ml/streptomycin 100 μg/ml (Sigma, Aldrich, Milano, Italy. P-0781), and then incubated for nine minutes at 37°C in a pre-warmed solution of PBS containing 2.4 U/ml dispase (Becton Dickinson and Company, Milano, Italy. 354235). After that, the fragments were digested with 0.05% trypsin/0.02% EDTA (Euroclone, ECB3052D) for 40 minutes at 37°C, in order to obtain AECs. The suspension was filtered using 80 μm filters (Millipore, Milan, Italy), and trypsin-undigested amnion fragments were digested again with pre-warmed 0.25% trypsin (Sigma, T-4049), strongly shaking for one to two minutes. After filtering again, trypsin in the suspension was neutralized with high glucose-Dulbecco’s modified Eagle’s medium (HG-DMEM; Euroclone, ECB7501L), supplemented with 10% heat-inactivated fetal bovine serum (FBS; Sigma Aldrich, F-7678) and AECs were collected by centrifugation at 200 x g for 10 minutes.

Cells were cultured in HG-DMEM supplemented with 10% FBS, 10 ng/ml epidermal growth factor (Sigma, E-9644), penicillin 100 UI/ml/streptomycin 100 μg/ml (Sigma, P-0781), 0.25 μg/ml amphotericin B (Sigma, A5955), 2 mM L-glutamine (Sigma, G-7513) and maintained at 5% CO_2_, and 38.5°C for the experiments described below. Medium was replaced after 72 hours for the first time to remove non-adherent cells and then it was replaced twice weekly or according to the experimental design. Adherent cells were detached with 0.05% trypsin/0.02% EDTA (Euroclone, ECB3052D) just prior to reaching plate confluence (80%) and then reseeded for culture maintaining. The cells were expanded for 10 passages.

### Proliferation assays: growth curve and doubling time analysis

To obtain the cell proliferation growth curve, cells at passage (P) 0 and P3 were seeded into six-well tissue culture dishes (Costar® Corning, NY, USA. 3516) at a density of 1 × 10^3^ cells/cm^2^. Every 2 days, through 14 days of culture, one well of the six-well dishes was trypsinized, and a cell count was performed. The number of viable cells was obtained by the Trypan blue dye exclusion method using a Burker chamber.

Doubling time (DT) analysis of AECs was also assessed. Culture passages from P1 to P10 were performed every four days and the number of viable cells for each passage was determined by the Trypan blue dye exclusion method using a Burker chamber. The population DT was obtained for each passage using the formula DT = CT/CD, where CT (culture time) is the time between passage “n” and passage “n + 1” and CD (cell doubling) = ln(Nf/Ni)/ln2, where Ni represents the seeded cells number and Nf the harvested cells number.

### Colony-forming unit assay

Colony-forming unit (CFU) assays were performed at P0 on freshly isolated cells at different densities (100, 250, 500 and 1,000 cells/cm^2^). Cells were plated in six-well plates and cultured in 5% CO_2_ and 90% humidity at 38.5°C for two weeks in HG-DMEM-supplemented medium. Then, colonies were fixed with 4% formalin and stained with 1% methylene blue (Serva, Heidelberg, Germany) in 10 mM borate buffer, pH 8.8 (Fluka BioChemika, Buchs, Swizerland) at room temperature, and washed twice. Colonies formed by 16 to 20 nucleated cells were counted under a BX71 microscope (Olympus Italia, Srl, Milano, Italy).

### RNA extraction and reverse transcription-polymerase chain reaction (RT-PCR)

Total RNA was extracted from feline amnion-derived cells at each passage, using TRIReagent (Sigma). Samples were then treated with DNAse (Sigma, D4263) in order to avoid DNA contamination. Both steps were performed according to the manufacturers’ specifications.

RNA concentration and purity were measured by Nanodrop Spectrophotometer (Nanodrop® ND1000 Thermo Scientific, Wilmington, USA). cDNA was synthesized from total RNA (500 ng) using Taqman Reverse Transcription reagents kit (Applied Biosystems, Life Tecnologies, Monza, Italy. 4304134).

Qualitative polymerase chain reaction (PCR) was performed in a 25 ml final volume with JumpStart Taq ReadyMix (Sigma-Aldrich, P2893) under the following conditions: initial denaturation at 94°C for 2 minutes, 32 cycles at 94°C for 30 seconds (denaturation), 55 to 60°C for 30 seconds (annealing), 72°C for 2 minutes (elongation) and final elongation at 72°C for 5 minutes.

The expression of the following set of genes was evaluated for molecular characterization before *in vitro* differentiation of cells: POU class 5 homeobox 1 (*POU5F1* alias *Oct4*), Nanog homeobox, as pluripotent-ESCs markers; phagocitic glycoprotein I (*CD44*), ALCAM (*CD166*), integrin beta-1 (*CD29*), 5′ nucleotidase ecto (*NT5E* alias *CD73*), thymus cell surface antigen theta-1 (*Thy1* alias *CD90*), as mesenchymal markers; Gp 105 to 120 (*CD34*), as hematopoietic marker; Major Histocompatibility Complex I and II (*MHC-I, MHC-II*), as immunogenic markers.

Feline specific primers were initially designed with the open source PerlPrimer software (v1.1.20, Parkville, Australia) based on NCBI *Felis catus* sequences or on mammal multi-aligned sequences, and subsequently manually improved. Primers were used at 200 nM final concentration.

To test *in vitro* cell differentiation, the following set of markers was used: bone gamma-carboxyglutamate osteocalcin (*OCN*) and osteopontin (*OPN*) for osteogenic differentiation; adiponectin (*ADIPQ*) and peroxisome proliferator-activated receptor γ (*PPAR-γ*) for adipogenic differentiation; aggrecan (*ACAN*) and collagen type 2 chain α1 (*COL2A1*) for chondrogenic differentiation; nestin (*NES*) for neurogenic differentiation. Feline mature tissues (bone, fat, cartilage and spinal cord) were used as positive controls for the expression of osteogenic, adipogenic, chondrogenic and neurogenic markers.

Glyceraldehyde 3-phosphate dehydrogenase (*GAPDH*) was used as reference a gene.

The sequences of each gene are shown in Table 1.

**Table 1:** Oligonucleotide sequences used for RT-PCR analysis

	***Gene***	***Primers***	***Product size***
**Housekeeping gene**	*GAPDH*	Forward, 5′ – ACGATGACATCAAGAAGGTG – 3′	180 bp
		Reverse, 5′ – CATACCAGGAAATGAGCTTG – 3′	
**Pluripotent markers**	*Oct4*	Forward, 5′ – GGAGTCCCAGGACATCAAAG – 3′	285 bp
		Reverse, 5′ – GCCTGCACAAGTGTCTCTGC – 3′	
	*Nanog*	Forward, 5′ – ACGGATCCAGCTCAGCCCCA – 3′	192 bp
		Reverse, 5′ – GGGGCTGCCCTGAGCAAGTA – 3′	
**Mesenchymal markers**	*CD44*	Forward, 5′ – TGGGTTGTTTGGCATCCAGTGC – 3′	100 bp
		Reverse, 5′ – CGTTTTCTTCAGTTGGTTCCCAGCC – 3′	
	*CD166*	Forward, 5′ – ACTGGCAGTGGAAGCGTCAT – 3′	275 bp
		Reverse, 5′ – CAGCAAGGAGGAGACCA – 3′	
	*CD29*	Forward, 5′ – GGAAACTTGGTGGCATTGTT – 3′	180 bp
		Reverse, 5′ – GTTCCTTGTAAACGGGCTGA – 3′	
	*CD73*	Forward, 5′ – AGCAAAGGGGCCACTAGCATCT – 3′	233 bp
		Reverse, 5′ – ACCCGAATGTCCCAGTGCAA – 3′	
	*CD90*	Forward, 5′ – GAGCACACGTACCGCTCCCG – 3′	233 bp
		Reverse, 5′ – AGCAGCAGCAGCAGCATCCA – 3′	
**Hematopoietic marker**	*CD34*	Forward, 5′ – CTTTAACTGTCACGGCGTTT – 3′	198 bp
		Reverse, 5′ – TGACTCGGGAACATTTGATT – 3′	
**Immunological markers**	*MHC-I*	Forward, 5′ – CATCACCCTGAGATGGGAGC – 3′	176 bp
		Reverse, 5′ – TGGGTACTGTCGTCGCGTG – 3′	
	*MHC-II*	Forward, 5′ – TCCGGAATCAGAAAGGACAC – 3′	172 bp
		Reverse, 5′ – GGCAAACCAAATCCTGAGAA – 3′	
**Osteogenic markers**	*OCN*	Forward, 5′ – CTGCCTCTGCCTGGCTGGTC – 3′	120 bp
		Reverse,5′ – TAGCGCCGGAGCCTCCTCAC – 3′	
	*OPN*	Forward, 5′ – ACTGGTCACTGATTTTCCCACGGA – 3′	100 bp
		Reverse, 5′– AACCACACTATCACCTCGGCCA – 3′	
**Adipogenic markers**	*ADPQ*	Forward, 5′ – TGAGAAAGGAGATCCAGGTC – 3′	308 bp
		Reverse, 5′ – TCAAGTAGACTGTGATGTGG – 3′	
	*PPAR-γ*	Forward, 5′ – CATGGTTGACACAGAGATGC – 3′	239 bp
		Reverse, 5′ – GCTCCACTTTGATTGCACTTTG –3′	
**Chondrogenic markers**	*ACAN*	Forward, 5′ – AAGTGGAGCCGCGTTTCCAAGG – 3′	163 bp
		Reverse, 5′ – AGTCATTGGAGCGCAGGTTCTGG – 3′	
	*COL2A1*	Forward, 5′ – AGTTGGGAGTAATGCAAG – 3′	294 bp
		Reverse, 5′ – GATAACCTCTGTGACCTTTG – 3′	
**Neurogenic marker**	*NES*	Forward, 5′ – AAACAGGGCCTACAGAG – 3′	293 bp
		Reverse, 5′ – ACAGGTGTCTCAAGGGTAG – 3′	

### *In vitro *multipotent differentiation

In order to test their multipotent differentiation potential, cells at P3 were seeded at a density of 1 x 10^3^/cm^2^ in six-well tissue culture dishes.

#### Osteogenic differentiation

For osteogenic differentiation, cells were cultured in HG-DMEM, supplemented with 10% FBS, 100 U/ml penicillin, 100 μg/ml streptomycin, 0.25 μg/ml amphotericin B, 2 mM/l L-glutamine, 10 mM β-glycerophosphate (Sigma, 50020), 0.1 μM dexamethasone (Sigma, D2915) and 250 μM ascorbic acid (Sigma, A8960). The osteogenic differentiation was assessed by incubating cells for up to three weeks at 38.5°C with 5% CO_2_. Non-induced control cells were cultured for the same time with standard medium (HG-DMEM supplemented with 10% FBS, 100 U/ml penicillin, 100 μg/ml streptomycin, 0.25 μg/ml amphotericin B, 2 mM/l L-glutamine). The medium was changed twice weekly. Presence of calcium deposits in differentiated cells was verified by von Kossa staining.

#### Adipogenic differentiation

For adipogenic differentiation, cells were cultured in HG-DMEM, supplemented with 10% FBS, 100 U/ml penicillin, 100 μg/ml streptomycin, 0.25 μg/ml amphotericin B, 2 mM/l L-glutamine, 10 μg/ml insulin (Sigma I-6634), 150 μM indomethacin (Sigma I-7378), 1 μM dexamethasone and 500 μM IBMX (3-isobutyl-methyl-xanthine, Sigma I-7018) and the adipogenic differentiation was assessed by incubating cells for up to three weeks at 38.5°C with 5% CO_2_. Non-induced control cells were cultured for the same time with standard medium. The medium was changed twice weekly. Differentiation was evaluated by Oil Red-O staining (Sigma, O0625), which dye intracytoplasmatic lipid droplets.

#### Chondrogenic differentiation

For chondrogenic differentiation, cells were cultured in DMEM low-glucose, containing 100 U/ml penicillin, 100 μg/ml streptomycin, 0.25 μg/ml amphotericin B, 2 mM/l L-glutamine, 100 nM dexamethasone, 50 μg/ml L-ascorbic acid 2-phosphate, 1 mM sodium pyruvate (BDH, Chemicals, Poole, UK. 44094BN), 40 μg/ml proline, ITS (insulin 5 μg/ml, transferrin 5 μg/ml, selenous acid 5 ng/ml; Sigma, I3146) and 5 ng/ml transforming growth factor-β3 (Peprovet, DBA Milano, Italy. 100-36E). The chondrogenic differentiation was assessed by incubating cells for up to three weeks at 38.5°C with 5% CO_2_. Non-induced control cells were cultured for the same time with standard medium. The medium was changed twice weekly.

Differentiation was evaluated by Alcian blue (Sigma, 89640) staining.

#### Neurogenic differentiation

The neurogenic differentiation was performed by incubating cells in a pre-induction medium consisting of HG-DMEM, 20% FBS and 1 mM β-mercaptoethanol (Sigma, M7522) for 24 h; then the neuronal induction was performed with a medium composed of HG-DMEM supplemented with 2% FBS, 2% dimethylsulfoxide (Sigma, D-5879) and 200 μM butylated hydroxyanisole (Sigma, B-1253) for three days. Non-induced control cells were cultured for the same time with standard medium. Differentiation was evaluated by Nissl staining to stain Nissl bodies.

The occurred differentiation was confirmed performing RT-PCR on undifferentiated (controls) and induced AECs (control cells) as described above.

### Detection of SSEA-3 and SSEA-4 markers by immunocytochemistry and flow cytometry

To test the expression of SSEA-3 and SSEA-4, as embryonic markers, primary rat and mouse antibodies respectively were purchased from Abcam (Cambridge, MA, USA), while Alexafluor-488 conjugated secondary antibodies were from Invitrogen (Carlsbad, CA, USA). All products were used following the manufacturer’s instructions.

#### Immunocytochemical characterization

For immunostaining, cells at P3 were fixed in 3.7% paraformaldehyde (PFA) for 15 minutes and washed three times in PBS. After that, cells were blocked using 2% bovine serum albumin (BSA) in PBS for 4 h at 4°C. Cells were incubated with primary antibodies overnight at 4°C. After washing three times, cells were incubated with rabbit anti-mouse and anti-rat Alexa Fluor 488-conjugated secondary antibody (1:250 dilution) for 1 h. Finally, for nuclear staining, cells were incubated for 15 minutes with Hoechst 33342 (1 mg/ml; Sigma) diluted 1:100 in PBS. The specificity of the immunostaining was tested by including negative controls, performed by use of non-immune mouse and rat serum (Santa Cruz Biotechnology Inc., Heidelberg, Germany) in place of specific antisera, and omission of the primary antibody.

Images were captured on a BX 51 microscope (Olympus, Japan).

#### Flow cytometry (FCM) analysis

At P3, AECs (2 x 10^6^ cells/ml) were labeled with primary antibodies for SSEA-3 and SSEA-4 in 3% BSA (BDH, VWR International Ltd., Poole, UK) in PBS for 45 minutes at room temperature in the dark. After that, cells were washed in cold PBS and incubated with secondary AlexaFluor-488 conjugated antibodies (1:250) for 30 minutes at room temperature in the dark. Labeled cells were washed twice in ice cold PBS and analyzed using an Epics Coulter flow cytometer (Beckman Coulter-IL, Fullerton, CA, USA). A minimum of 10,000 cells were acquired for the evaluation of each antibody and analyzed in the FL1 channel. All analyses were based on control cells incubated with isotype-specific IgG or IgM to establish the background signal. Files analysis was performed using Weasel software v.2.5 available online (Parkville, Australia) [46].

### Statistical analysis

Statistical analysis was performed using GraphPad Instat 3.00 for Windows (GraphPad Software, La Jolla, CA, USA). Three replicates for each experiment (growth curves, doubling times and CFU) were performed and the results are reported as mean standard deviation (SD). One-way analysis of variance (ANOVA) for multiple comparisons by Student-Newman-Keuls multiple comparison tests was used. CFU comparison among different cell plating densities inside each group was analyzed*. P <*0.05 was considered as significant.

## Results

### Amnion collection and isolation of amniotic epithelial cells

Cells adhered to culture dishes and in the first culture displayed initial morphological heterogeneity with epithelial, fibroblastic-like and circular cells. Subsequently, a typical polygonal epithelial morphology was recognized. Moreover, clusters of rapidly expanding cells were observed.

**Figure 1:**
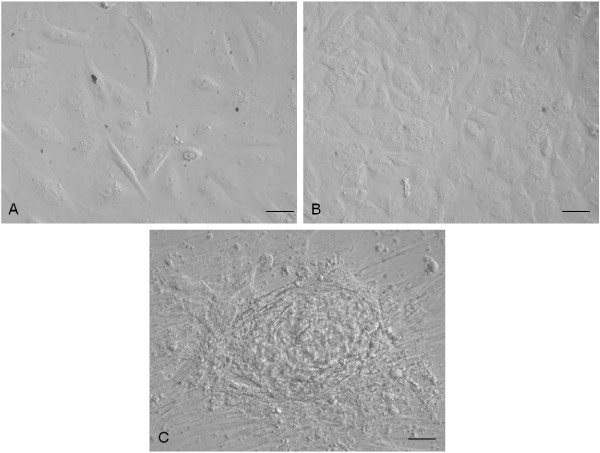
**Cell morphology. (A)** Monolayer of cells in first culture and **(B)** at passage 3 (P3); **(C)** Amniotic epithelial cells (AECs) with cluster. Magnification 20×; scale bar = 20 μm.

Representative images are shown in Figure 1.

### Proliferation assay: growth curve and doubling time analysis

**Figure 2:**
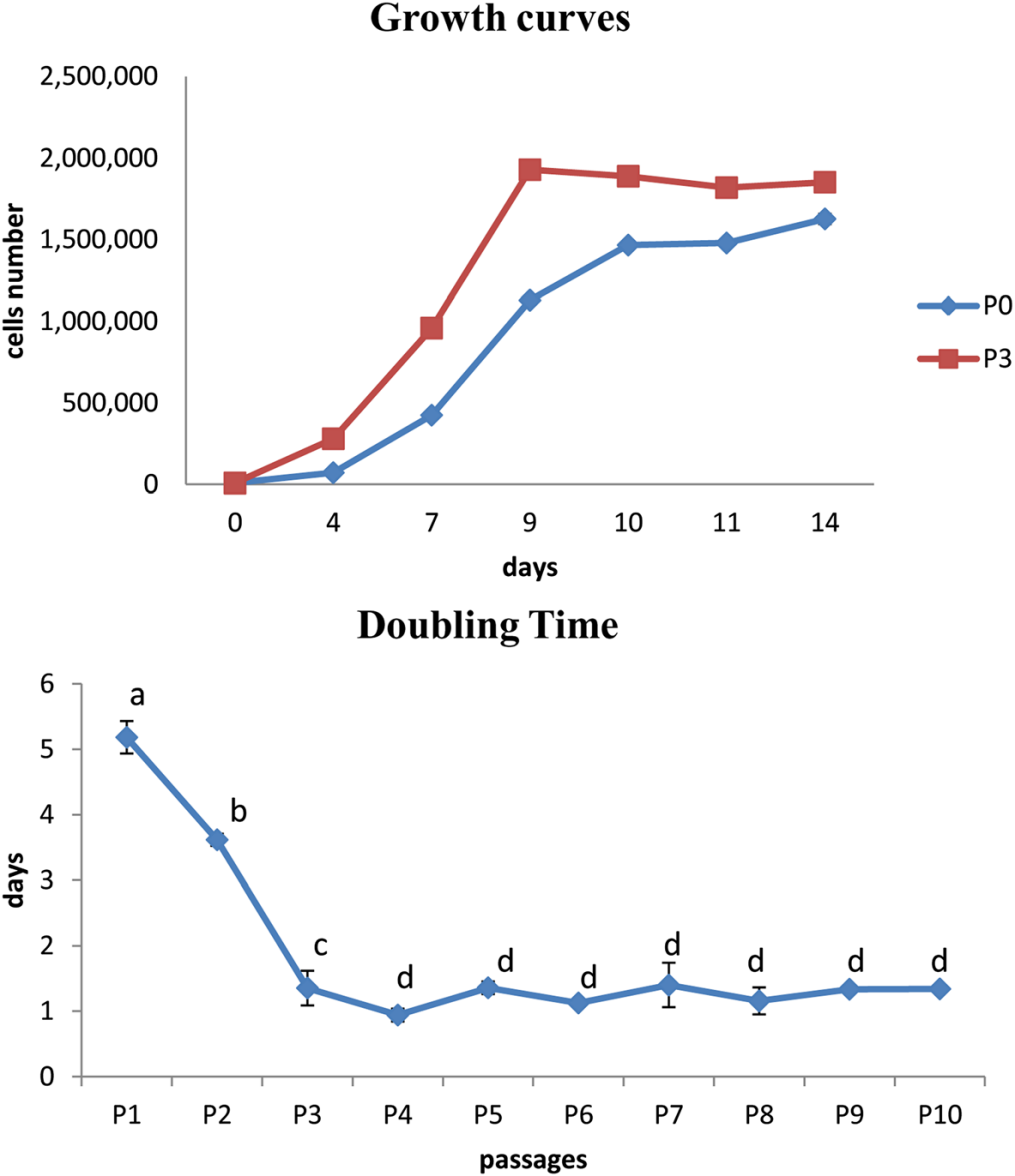
**Proliferation assay.** AECs growth curve at P1 and P3; doubling times at different passages during cell culture. Letters represent doubling time means statistically different. **a**, **b**: *P* <0.05; **c**, **d**: *P* <0.01.

Studying the growth curve of feline AECs it was possible to observe at P0 a slow plating efficiency with a lag phase of 48 hours compared to cells at P3 that showed an immediate and intensive log phase until the ninth day. In both curves there was a final plateau phase from the 9^th^ to the 14^th^ day (Figure 2).

At P1 and P2, the population DT was higher compared to the other passages, with mean values of 5.2 ± 0.25 and 3.6 ± 0.09 days, respectively. From P3 to P10, the mean DT value was 1.25 ± 0.14 days. Differences were statistically significant comparing P1 with P2 (*P* <0.05) and highly significant comparing P1 with the subsequent passages (*P* <0.01) (Figure 2).

### Colony-forming unit assay

The number of cell colonies formed was counted at P0 after seeding cells at different density/cm^2^. AECs demonstrated a statistically significant increase in CFU frequency with increasing cell-seeding densities (Table 2).

**Table 2:** CFU assay

	**Density cells/cm**^**2**^	**Total cells**	**CFU**	**1 CFU each**
**AECs**	100	950	1.82 ± 0.72^a^	521.98
	250	2,375	15.93 ± 1.39^b^	149.09
	500	4,750	20.54 ± 2.72^c^	231.25
	1,000	9,500	37.43 ± 2.67^d^	253.81

Different small letters superscripts (a, b, c, d) indicate statistically different comparisons (*P* <0.05) between cell densities in each group (amniotic epithelial cells (AECs)).

### RNA extraction and RT-PCR analysis

**Figure 3:**
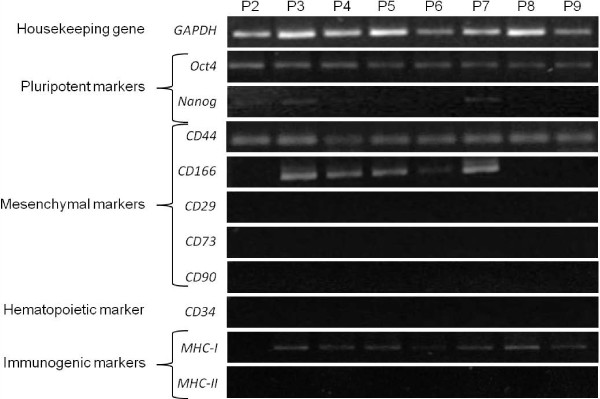
**RT-PCR analysis.** Pluripotent (*Oct4* and *Nanog*), mesenchymal (*CD44, CD166, CD29, CD73, CD90*) and hematopoietic (*CD34*) specific gene expression on AECs from P1 to P9. Major Histocompatibility Complex (*MHC*) I and II gene expression is also reported. *GAPDH* was used as reference gene. AECs, amniotic epithelial cells.

As shown by RT-PCR, AECs expressed the pluripotency-associated markers *Oct-4* and *Nanog*: mRNAs for *Oct-4* were detected over the passages studies, whereas those for *Nanog* were only expressed at the first passages (P2 and P3) and surprisingly at P7. Cells also showed expression for some of the mesenchymal stem cell- (MSC-)associated markers (being positive for *CD44* at each passage and for *CD166* from P3 to P7) and lacked of the hematopoietic marker *CD34*. Expression for other MSC-specific markers (*CD29*, *CD73* and *CD90*) has not been registered at any stage. *MHC-I* expression was demonstrated in each cell population, whereas *MHC-II* was not. Figure 3 shows the expression of the specific genes evaluated.

### *In vitro* multipotent differentiation

#### Osteogenesis

After three weeks of culture in osteogenic induction medium, feline AECs distinctly changed their morphology and were surrounded by calcium deposits positive to von Kossa staining. In controls, cells did not change in morphology and did not stain positively to von Kossa.

**Figure 4:**
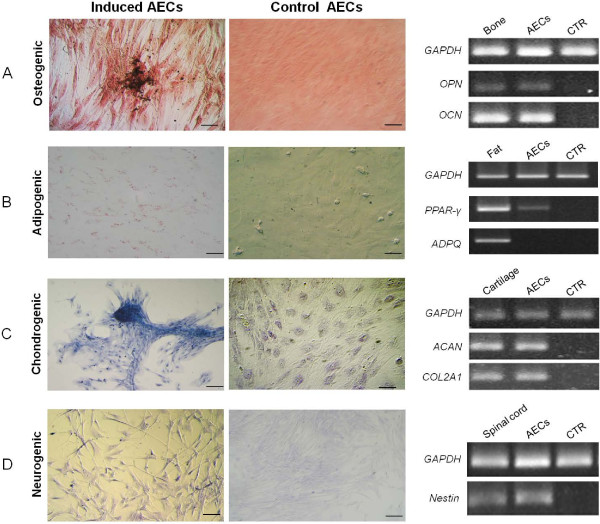
**Staining of differentiated and control undifferentiated feline AECs and respective molecular expression. A)** von Kossa staining after osteogenic induction and RT-PCR analysis of osteopontin (*OPN)* and osteocalcin (*OCN)*. **B)** Oil Red-O positive cytoplasmic neutral lipids after adipogenic induction and RT-PCR analysis of *PPAR-*γ and adiponectin (ADPQ). **C)** Alcian blue staining after chondrogenic induction and RT-PCR of aggrecan (*ACAN*) and collagenase (*COL2A1)*. **D)** Nissl staining after neurogenic induction and RT-PCR of nestin. Magnification 20×; scale bar = 20 μm. *GAPDH* was employed as a reference gene. Bone, adipose tissue, cartilage and spinal cord were used as positive controls. AECs, amniotic epithelial cells.

The osteogenic differentiation was confirmed by the expression of *OPN* and *OCN* mRNAs. A weak expression of *OCN* was also registered in the controls (Figure 4A).

#### Adipogenesis

In cells induced to differentiate into adipocytes, the presence of intracytoplasmatic lipids droplets was evident after three weeks, whereas control cells showed no lipid deposits. Molecular analysis confirmed the induction revealing the expression of *PPAR-γ*, whereas mRNAs for adiponectin were not detected (Figure 4B).

#### Chondrogenesis

Cells grown in chondrogenesis-inducing medium stained positively for Alcian Blue, demonstrating a marked deposition of metachromatic extracellular matrix composed by glycosaminoglycans. Controls did not change in morphology and were negative to Alcian blue staining. RT-PCR showed that differentiated cells express *ACAN* and *COL2A1*, confirming the induction (Figure 4C).

#### Neurogenesis

When induced into neurogenic lineage, cells showed an increased presence of Nissl bodies and displayed the typical neuronal morphology with axon- and dendrite-like processes, as compared to the polygonal epithelial morphology observed in cells cultured in basal conditions. RT-PCR confirmed the neurogenic differentiation (Figure 4D).

#### Detection of SSEA-3 and SSEA-4 markers by immunocytochemistry and flow cytometry

**Figure 5:**
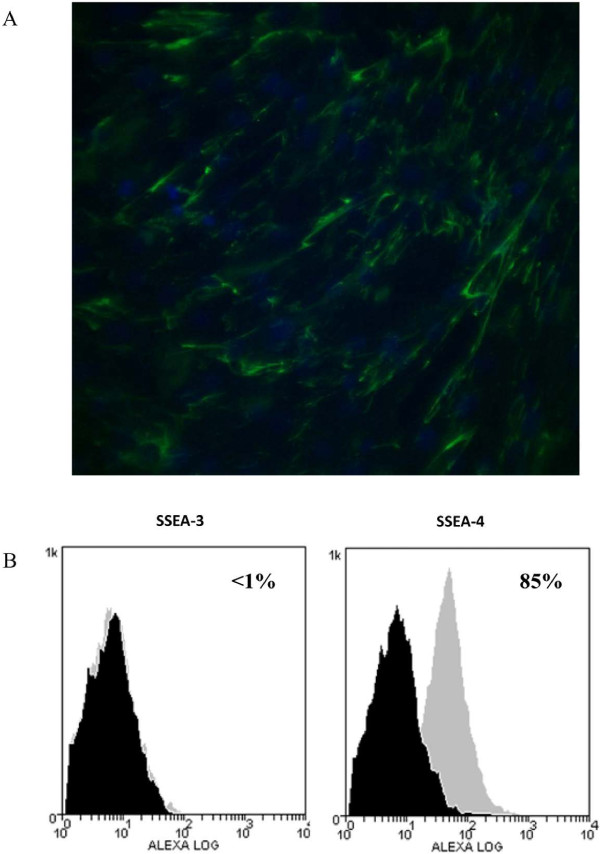
**Immunostaining and cytometry analyses of SSEA-3 and SSEA-4 antigens. A)** Photomicrographs of immunostaining of feline amniotic epithelial cells (AECs). Cells labeled with antibodies against antigen SSEA-4. Magnification 20×, scale bar = 20 mm. **B)** Flow cytometry analysis of SSEA-3 and SSEA-4 antigen expression with Alexafluor-488 labeled antibodies. Histograms represent relative number of cells vs. fluorescence intensity (FL1). Black histograms indicate background fluorescence intensity of cells labeled with isotype control antibodies only; gray histograms show positivity to the studied antibodies.

Only immunopositivity to the stem cell markers SSEA-4 was detected. AECs expressed this antigen on the cell surface (Figure 5A).

FCM analysis revealed that AECs were negative for SSEA-3 while 85% of cells showed SSEA-4 reactivity (Figure 5B).

## Discussion

The identification of the optimal source of stem cells represents a critical issue for cell therapy, in order to obtain a relevant amount of cells and to minimize risks for the donors and the recipients. For these purposes, amniotic membrane is a valid alternative, in particular for the isolation of epithelial cells [23,27,36,37]. Despite the importance of the domestic cat in studying human genetic and viral diseases, to our knowledge no studies have been performed on feline amniotic epithelial cells.

In the present study, for the first time, we have isolated and expanded AECs from feline amnion that is an extra-fetal tissue and thus retains higher proliferation and differentiation potential respect to cells deriving from adult compartments. Cells, easily isolated through enzymatic digestion, showed typical polygonal epithelial morphology and were able to be sub-cultured *in vitro*.

The proliferation study (growth curve) showed that AECs reached high plating efficiency at P3, as demonstrated by the short lag phase in respect to P0. This result was probably due to the fact that the first culture is composed of a heterogeneous cell population that possesses a lower potential, as MSCs. According to this, but in contrast with data reported in literature [36,38], the DT analysis revealed a high value for the first passages (P1: 5.19 ± 0.25 days; P2: 3.62 ± 0.09 days). To confirm our hypothesis, on the other hand, the initial heterogeneity of cell population was also recognized morphologically during the primary culture (at P0), when fibroblastic-like and circular cells were present. By the first passage, a selection of typical polygonal epithelial cells occurred and from P3 to P9 the proliferation rate increased with a mean DT value of 1.24 ± 0.17 days, suggesting that at P3 AECs reached the sufficient levels of homogeneity. This indicates the higher proliferating ability of the isolated AECs, in accordance with Miki *et al*. [38] and Parolini *et al*. [36] in humans, and with Lange-Consiglio *et al*. [27] in equine species, who reported robust proliferation at least up to P6.

When AECs were seeded at different densities, they were able to form clones with frequency that increased with the cell-seeding density, suggesting that paracrine signaling between cells at P0 occur [47]. Moreover, when AECs are kept in high-density cultures, small cell clusters or spheroid structures developed, showing that these cells did not have contact-inhibited cell growth and continue to proliferate after reaching 100% surface confluence, forming aggregates overlying the monolayer of confluent cells. Miki *et al*. [32,34,38] report that the amniotic cells in monolayer may support the growth and maintain undifferentiated the amniotic cells of spheroid structures, possibly playing the role of an autologous feeder layer and providing secreted factors.

Molecular characterization by RT-PCR showed the expression of some of the pluripotency-associated transcription factors, as *Oct4* at each of the passages studied, in agreement with data reported in previous studies [26,27,32-34,36,37,39,42]. In particular, *Oct4* is known to play a critical role in maintaining pluripotency and self-renewal of the epiblast and it is down-regulated during gastrulation, suggesting that amniotic epithelium could maintain the potency of undifferentiated epiblast [32]. *Nanog* was only expressed at the first passages (P2 and P3) and surprisingly at P7. These data could be due to the relative heterogeneity of cells and/or to the changes in membrane expression markers that may occur from one culture passage to another, as observed by Corradetti *et al*. [48] in the horse. AECs did express *CD44* and *CD166* from P3 to P9 but lacked the expression of other mesenchymal markers, as *CD29*, *CD73* and *CD90*, confirming the heterogeneity observed by the proliferation studies. Bilic *et al*. [48,49] reported that human AECs had an antigen expression profile characteristic of culture-expanded MSCs and could co-express epithelial and mesenchymal cell markers [50]. In fact, it has been reported that the amnion-derived cells have not completely differentiated into epithelial or mesenchymal phenotype, or another explanation is that the epithelial-mesenchymal transition may occur in the amniotic membrane [49,50]. Expression of the hematopoietic marker *CD34* was not found at any passage. This result was expected, and it confirmed that isolated cells do not belong to a hematopoietic lineage. Furthermore, isolated cells seem to be immune-privileged, as confirmed by the expression of *MHC-I* and the absence of *MHC-II* expression over the passages studied. The lack of this marker hints at potential application of these cells to allo- and xeno-transplantation, in agreement with previous studies [27,32,33,36,37,39,42,51].

It is important to underline that RT-PCR alone is not useful for characterizing AECs and that quantitative analysis are needed to make meaningful statements about their gene expression. The investigation with flow cytometry provides useful quantitative data on the percentage of reactivity, but, as reported by Iacono *et al*. [30], there are no commercially available feline species-specific antibodies for characterization of these MSCs.

In addition to molecular characterization by RT-PCR, we tried to detect the immunopositivity of amniotic feline epithelial cells to SSEA-3 and SSEA-4 that are cell surface globo-series glycosphingolipid epitopes that are commonly used as markers for human embryonic stem cells [52,53]. Lately, SSEA-3 and -4 have also been observed in MSCs from different origin [54-57]. Our data showed that 85% of cultured cells display SSEA-4 on their surface but not SSEA-3. One interpretation of these findings could be that amnion-derived cells have a subset of primitive stem cells. In this regard, it is noteworthy that the epithelial layer of horse amnion has the same epiblastic origin [58] as the human layer, and it is therefore reasonable to speculate that some AECs may have escaped the specification that accompanies gastrulation, and that these cells may retain some or all of the characteristics of epiblastic cells, such as pluripotency [32]. In contrast to SSEA-4, SSEA-3 epitope was not detected in feline AECs. Probably this result is not a surprise because it is known that SSEA-3 is extinguished more rapidly from the cell surface than SSEA-4 during embryonic stem-cell differentiation [59,60].

Further investigation will be required in order to determine whether the amniotic cells, positive to some stem cell marker, are remnants of the pluripotent cells from the fetus or if amniotic cells maintain their stem cell nature for a separate and specific function which has yet to be determined. As reported by Miki and Strom [32], if placental stem cells are maintained throughout the pregnancy, the mechanism and the functional implications of this will be the basis of future exploration.

In an effort to determine the plasticity of AECs, *in vitro* differentiation assays have been performed and despite the lack of expression of some stem cell marker, it was possible to define feline AECs as presumptive stem cells. *Nes* expression, the Nissl staining and the changes in morphology observed suggested neuronal differentiation of the cells when kept under neurogenic culture condition, confirming their ability to differentiate into the ectodermal lineage. Furthermore, under *in vitro* induction conditions, we were able to differentiate feline AECs toward two mesodermal lineages, such as osteocytes and chondrocytes. These data were confirmed by specific gene expression analysis and specific stainings, and are in accordance with those previously reported for human, equine and ovine AECs [26,27,33]. When stimulated to differentiate toward the adipogenic lineage, however, AECs expressed only mRNA for *PPAR-*γ that is crucial for the pre-adipocyte commitment [61]. The lack of expression of adiponectin, that is a collagen-like protein exclusively synthesized in white adipose tissue [62], might be led to the culture conditions employed in this study or to some specific characteristics of the feline AECs that need more time for adipogenic differentiation compared to other cell lines. Other extra-fetal cell lines, as umbilical cord blood (UCB)-derived stem cells, present much less obvious adipogenic differentiation than bone marrow- or adipose-derived MSCs [63,64]. Bieback *et al*. [65] did not obtain adipocytes after culture of human UCB cells in standard induction medium, containing dexamethasone, IBMX, insulin, indomethacin and FBS. However, continuous culture in induction medium for five weeks did result in some adipogenic differentiation. Similarly, only sporadic fat cells containing limited amounts of lipid droplets were evident in equine UCB cell cultures after 21 days in adipocyte induction media [66]. Lee *et al*. [67] achieved adipogenic differentiation but only after the addition of rabbit serum to the induction medium. Kern *et al*. [12] reported a failure of human UCB cells to induce differentiation into adipocytes, even following five weeks of culture.

In the effort to check pancreatic differentiation (endodermic lineages), by different protocols, differentiation was not obtained in this cell line, in our opinion due mainly to technical problems (see Additional file 1). Since these cells differentiated into two germ layers (mesodermic and ectodermic), feline AECs could be defined as multipotent, unlike human amniotic epithelial stem cells that showed pluripotency by the ability to differentiate into all three germ layers [34].

## Conclusions

In conclusion, although BM-derived cells have received the most attention and are thus the best characterized, the procedure to isolate these cells requires significant time in culture and the recovery efficiency is low. This lag time prevents earlier use of these cells, which may prove to be suboptimal. AECs have no lag time in culture as they are isolated and cultured at birth or during ovario-hysterectomy. Moreover, these cells are capable of differentiation into two germ lines and have low immunogenicity, making them an ideal candidate for allogeneic implantation.

From the results obtained, it is possible to say that feline amniotic membrane, that could be collected at the delivery, during caesarean section or after ovariohysterectomy of pregnant queens, may be considered as a remarkable source of multipotent stem cells in cats, available for future efforts in cell therapy. However, further studies, including pre-clinical, and a deeper evaluation of stemness properties (as pancreatic differentiation, telomerase activity, clonal expansion, unrestricted growth, teratoma formation in mice) are needed for the *in vivo* applications in order to better understand their applicability for tissue regeneration *in vivo* and immune host reaction.

## Abbreviations

AEC: Amniotic epithelial cell; BM: Bone marrow; BSA: Bovine serum albumin; CFU: Colony forming unit; DT: Doubling time; FBS: Fetal bovine serum; FCM: Flow cytometry; HG-DMEM: High glucose–Dulbecco’s modified Eagle’s medium; IBMX: 3-isobutyl-methyl-xanthine; MHC: Major histocompatibility complex; MSC: Mesenchymal stem cell; P: Passage; PBS: Phosphate buffered saline; RT-PCR: Reverse transcription-polymerase chain reaction; UCB: Umbilical cord blood.

## Competing interests

The authors declare that they have no competing interests.

## Authors’ contributions

LR carried out the isolation and differentiation of cells, participated in molecular genetic studies and the sequence alignment, and drafted the manuscript. BC carried out the molecular genetic studies and the sequence alignment and helped in drafting the manuscript. LV and DB participated in the design of the experiments and helped in drafting the manuscript. AM participated in the isolation and differentiation of cells, and in molecular genetic studies, and sequence alignment. FC conceived of the study, participated in its design and performed the surgeries for amnion collection. ALC conceived of the study, designed and coordinated the experiments, performed the statistical analysis and drafted the manuscript. All authors have read and approved the final manuscript.

## Supplementary Material

Additional file 1 **Pancreatic differentiation.** Supplementary information on protocols, results and discussion regarding the experiments aimed to evaluate the endodermic lineage differentiation of feline progenitor like amniotic epithelial cells. Click here for file
