# Supplementary material for: Molecular characterization and in vitro differentiation of feline progenitor-like amniotic epithelial cells
Source: Stem Cell Res Ther. 2013 Oct 30;4(5):133. doi: 10.1186/scrt344 (PMC3854755; doi:10.1186/scrt344)
Supplement: Additional file 1 — Pancreatic differentiation. Supplementary information on protocols, results and discussion regarding the experiments aimed to evaluate the endodermic lineage differentiation of feline progenitor like amniotic epithelial cells. [file scrt344-S1.pdf]

PANCREATIC DIFFERENTIATION

MATERIALS AND METHODS

Pancreatic differentiation was performed by two different protocols. In the first protocol, cells were cultured for 14 days in HG-DMEM supplemented with 10% FBS, 100 U/ml penicillin, 100 µg/ml streptomycin, 0.25 µg/ml amphotericin B, 2 mM/l L-glutamine, 1% nonessential amino acid, 55 µM 2-mercaptoethanol (Sigma, M-3148), 1 mM sodium pyruvate and 10 mM nicotinamide (Sigma, N-0636) [1].

In the second protocol, $1x10^5$ AECs were seeded onto a collagen I-coated 60 mm plastic dish (Corning CellBIND surface, CC3294) and cultured in the growth medium described above. When the cells reached 100% confluence, a "three-step" protocol to induce pancreatic cell differentiation was used. In the first step, the medium was replaced with HG-DMEM supplemented with 10% FBS and 10^{-6} M retinoic acid (Sigma, R-2625) and cultured for 2 days; the medium was then replaced with high HG-DMEM supplemented with 10% FBS alone and cultured for 2 days. In the second step, the cells were dissociated by PBS supplemented with 0.25% EDTA, plated in a Matrigel matrix six well plate (Euroclone, Reinnervate, REAVP0043X2), and cultured for 7 days in low glucose DMEM supplemented with 10% FBS, 10 mM nicotinamide, 20 ng/ml hEGF, and 1xN2 supplement (Lyfe Technology, MB, Italy, 17502048). In the third step, the medium was replaced with low glucose DMEM supplemented with 10 nM exendin-4 (Sigma, E-7144) and the cells were cultured for 7 days [2].

Differentiation was evaluated by morphological changes and by RT-PCR for insulin *(INS)*, glucagon *(GCG)* and somatostatin *(SST)*. Feline pancreas were used as positive control for the expression of pancreatic markers, while glyceraldehyde 3-phosphate dehydrogenase (*GAPDH*) was used as reference gene.

	Gene	*Primers*	*Product size*
**Housekeeping gene**	*GAPDH*	Forward, 5' – ACGATGACATCAAGAAGGTG – 3'<br>Reverse, 5' – CATACCAGGAAATGAGCTTG – 3'	180bp
**Pancreatic markers**	*INS*	Forward, 5' – TAACCAGCACCTTTGTGGCT – 3'<br>Reverse, 5' – CACAGCATTGCTCCACGATG – 3'	212bp
	GCG	Forward, 5' – AGTTCCGAAAAGAGGGCTCG – 3'<br>Reverse, 5' – GAATTCCTTGGCAGCTTGGC – 3'	426bp
	SST	Forward, 5' – TGCTGTCTGAACCCAACCAG – 3'<br>Reverse, 5' – TGGATCTCTGCAGTTCCAGC – 3'	102bp

RESULTS

After induction of pancreatic differentiation by two different protocols, AECs did not change their epithelial morphology and the expression of pancreas specific genes was negative, suggesting that these culture treatments did not induce pancreatic differentiation of AECs.

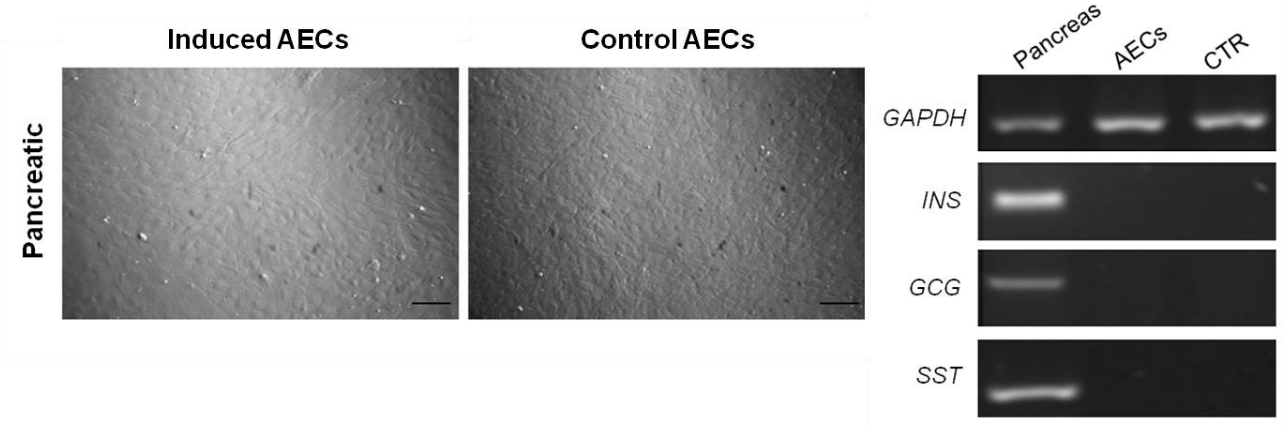

DISCUSSION

In the effort to check pancreatic differentiation (endodermic lineages), performing this experiment with different protocols, differentiation was not obtained in this cell line, in our opinion due mainly to technical problems. Since cells differentiated in two germ layers (mesodermic and ectodermic), feline AECs could be defined as multipotent, unlike human amniotic epithelial stem cells that showed pluripotency by the ability to differentiate into all three germ layers [1].

BIBLIOGRAPHY

1. Miki T, Lehmann T, Cai H, Stolz DB, Strom SC: **Stem Cell Characteristics of Amniotic Epithelial Cells**. *Stem Cells* 2005, 23(10):1549-1559.

2. Tamagawa T, Ishiwata I, Sato K, Nakamura Y: **Induced in vitro differentiation of pancreatic-like cells from human amnion-derived fibroblast-like cells**. *Human Cell* 2009, 22(3):55-63.
